# Integration of AI-2 Based Cell-Cell Signaling with Metabolic Cues in *Escherichia coli*

**DOI:** 10.1371/journal.pone.0157532

**Published:** 2016-06-30

**Authors:** Arindam Mitra, Christopher D. Herren, Isha R. Patel, Adam Coleman, Suman Mukhopadhyay

**Affiliations:** Virginia-Maryland Regional College of Veterinary Medicine, University of Maryland, College Park, Maryland, United States of America; University of Maryland, College Park, UNITED STATES

## Abstract

The quorum sensing molecule Autoinducer-2 (AI-2) is generated as a byproduct of activated methyl cycle by the action of LuxS in *Escherichia coli*. AI-2 is synthesized, released and later internalized in a cell-density dependent manner. Here, by mutational analysis of the genes, *uvrY* and *csrA*, we describe a regulatory circuit of accumulation and uptake of AI-2. We constructed a single-copy chromosomal *luxS-lacZ* fusion in a *luxS* ^*+*^ merodiploid strain and evaluated its relative expression in *uvrY* and *csrA* mutants. At the entry of stationary phase, the expression of the fusion and AI-2 accumulation was positively regulated by *uvrY* and negatively regulated by *csrA* respectively. A deletion of *csrA* altered message stability of the *luxS* transcript and CsrA protein exhibited weak binding to 5’ *luxS* regulatory region. DNA protein interaction and chromatin immunoprecipitation analysis confirmed direct interaction of UvrY with the *luxS* promoter. Additionally, reduced expression of the fusion in *hfq* deletion mutant suggested involvement of small RNA interactions in *luxS* regulation. In contrast, the expression of *lsrA* operon involved in AI-2 uptake, is negatively regulated by *uvrY* and positively by *csrA* in a cell-density dependent manner. The dual role of *csrA* in AI-2 synthesis and uptake suggested a regulatory crosstalk of cell signaling with carbon regulation in *Escherichia coli*. We found that the cAMP-CRP mediated catabolite repression of *luxS* expression was *uvrY* dependent. This study suggests that *luxS* expression is complex and regulated at the level of transcription and translation. The multifactorial regulation supports the notion that cell-cell communication requires interaction and integration of multiple metabolic signals.

## Introduction

Quorum sensing is a process of cell-to-cell communication in bacteria via freely diffusible molecules called autoinducers, which modulates gene expression in a population density-dependent manner [1, 2]. Many physiological processes and group behaviors such as motility, swarming, exopolysaccharide synthesis, stress survival, biofilm formation and virulence in bacteria are mediated by quorum sensing (QS) [2–8]. Interference with quorum sensing by quorum sensing inhibitors (QSI) can block infection processes and consequently have the potential to tackle infectious disease caused by antibiotic resistant pathogens [9]. *E*. *coli* is known to synthesize at least three types of autoinducers of which autoinducer-2 (AI-2) is generated as a byproduct of the activated methyl cycle and requires the action of enzyme, LuxS in a key step of the process[10, 11].The gene *luxS* encodes S- ribosyl homocysteinase that interconverts S-ribosyl homocysteine to homocysteine generating a furnanone borate ester, the active autoinducer, AI-2. AI-2 is thought to be a metabolic cue as it is generated from a central metabolic pathway. Homologs of *luxI*, the AI-1 synthase, are not found in *E*. *coli* and consequently Acyl-homoserine lactone (AI-1) is not detected in cell-free supernatant from cultures of *E*. *coli*, however a AI-1 receptor is present [11]. The *luxS* gene is conserved across many gram-positive and gram-negative bacterial pathogens and it is thought to be acquired by horizontal transfer million years ago [12–14]. Consequently synthesis of AI-2 is thought to be universal across both gram-positive and gram-negative bacterial species and thus AI-2 is considered as universal signaling molecule. Accumulation of AI-2 is controlled by a homolog of ribose uptake transport system, Lsr, (*luxS* regulated), which imports AI-2 from the external environment. Induction of the Lsr system at high cell density minimizes the levels of AI-2 from the extracellular milieu [15, 16]. Several quorum sensing circuits are well established in many clinically important pathogens, many of which integrate with two-component regulatory systems [17–21].

Two-component regulatory systems (TCS) are unique bacterial signaling systems that facilitate adaptation in a rapidly changing environment [22–25]. TCS consists of a sensor kinase that senses and transmits external signals to its cognate response regulator by phosphorelay; the response regulator upon phosphorylation regulates gene expression usually by transcription activation [26]. TCS are attractive choice as drug targets for pathogenic bacteria as several of them are strongly are associated with virulence [27]. *E*. *coli* harbors more than thirty two-component regulatory systems, many of which are linked with pathogenesis [28]. The fimbrial gene regulations are an important contributing factor in virulence and establishing an infection. Previously, we have elucidated regulation of biofilm formation, adhesion, motility and virulence genes by an important two-component regulatory system, the BarA/UvrY/CsrA pathway in extra-intestinal pathogenic *Escherichia coli*. Particularly, we found that in avian pathogenic *Escherichia coli* (APEC) and uropathogenic *Escherichia coli* (UPEC), *uvrY* stimulates transcription of fimbrial and virulence genes [29–31]. Because the BarA/UvrY/CsrA pathway is strongly associated with virulence, we hypothesized that QS might be one of the mechanism by which virulence and other pleiotropic roles could be mediated through the pathway in *E*. *coli*.

Several other observations led us to hypothesis about the association of this TCS and AI-2 based quorum sensing. Similar to the association of virulence with the BarA/UvrY/CsrA TCS pathway, the *luxS* gene is also linked to pathogenesis of Enteropathogenic and Enterohemarrhagic *E*. *coli* [32, 33]. Our previous studies have also demonstrated that *luxS* contributes to pathogenicity in APEC [34]. Furthermore, UvrY is a LuxR type transcriptional regulator which is commonly associated with quorum sensing [35]. Expression of small RNA CsrB and CsrC small RNAs are under the positive control of the BarA-UvrY-CsrA pathway in a cell density mediated manner. Phenotypes such as biofilm formation, swarming motility and virulence associated with the BarA-UvrY-CsrA pathway are dependent on community associations of microbes [31]. Most importantly, both the BarA/UvrY/CsrA pathway and AI-2 based quorum sensing contributes to biofilm formation in *E*. *coli* [36]. These observations led us to study the regulatory effect of the pathway on *luxS* based quorum sensing. In this study, we investigated the role of the *uvrY* and *csrA* genes in regulation of synthesis and uptake of AI-2 and *luxS* gene expression. Based on our results, we propose a regulatory circuit that controls quorum sensing at the transcriptional level via *uvrY* and post transcriptional level via CsrA. These findings suggest a principal role of BarA-UvrY-CsrA system in establishing early infection in pathogenic proteobacteria via quorum sensing and mediating a switch from a planktonic state to biofilm mode of persistence.

## Materials and Methods

### Bacterial strains, plasmids, media and growth conditions

Bacterial strains, plasmids, and bacteriophages used in this study are listed in Table 1. *E*. *coli* was grown in Luria-Bertani (LB) medium (10 gl^-1^ Tryptone, 5 gl^-1^ Yeast Extract, 10 gl^-1^ Sodium Chloride, pH 7) and strains harboring λ fusions were grown in Tryptone Broth (TB) (10gl^-1^ Tryptone, 5gl^-1^ Sodium Chloride, pH 7). Selection of phage λ lysates and plating’s were done on R medium (10 gl^-1^ tryptone, 1 gl^-1^ yeast extract, 5 gl^-1^ NaCl, 1 mM CaCl_2_ and 0.1% glucose). M9 minimal medium with or without 0.1% Casamino acids was used for glucose induction assay. *V*. *harveyi* strains were grown in AB medium (17.5 gl^-1^ NaCl, 12.3 gl^-1^ MgSO_4_, 2 gl^-1^ Casamino acids, pH 7.5) supplemented with 10 mM potassium phosphate (pH 7.0), 1 mM L-arginine and 1% glycerol. The antibiotics were added in the given concentration; Ampicillin 100 μg ml^-1^, Chloramphenicol 20 μg ml^-1^, Kanamycin 50 μg ml^-1^, Streptomycin 50 μg ml^-1^ and Tetracycline 10 μg ml^-1^. For proper growth, all strains were grown in baffled flasks at 150 rpm in shaking water bath at 37°C or 30°C. For gene expression experiments, overnight cultures were diluted 1:100 and subcultured two times to an OD_600_ of 0.30, before inoculation into fresh pre-warmed media to an initial OD_600_ of 0.05.

**Table 1:** List of bacterial strains and plasmids used in the study.

Strains or plasmids	Genotype of strains or function of plasmids	Source or Parent
MG1655	F^-^ λ^-^ *ilvG rbf50 rph1*	F. Blattner
MG1655Δ*lac*	F^-^ λ^-^ *ilvG rbf50 rph1lac*	D. J. Jin
DH5α	*luxS supE44 Δ (Φ80 ΔlacZ M15) hsdR17 recA1 endA1 gyrA96 thi-1 relA1*	Invitrogen
SP850	Hfr *λ*^-^ *e14- relA1 spoT1 thiE1 ΔcyaA1400(*::*kan)*	Coli Genetic Stock Center
AKPO14	MC4100Δ*barA*::*kan*	[35]
CB369	Δ*crp*::*kan*	T. Conaway/MG1655
RG1B-MG1655	Δ*csrB*::*cam*	[51]/MG1655
TR1-5 MG1655	Δ*csrA*::*kan*	[51]/MG1655
SM1000	Δ*barA*::*kan*	MG1655 Δ*lac*
SM1002	Δ*uvrY*::*cam*	MG1655 Δ*lac*
SM1003	Δ*csrA*::*kan*	MG1655 Δ*lac*
SM1005	MG1655 Δ*lac att*::λΦ(*luxS’-‘lacZ*)	MG1655 Δ*lac*
SM1006	Δ*barA*::*kan*	SM1005
SM1007	Δ*uvrY*::*cam*	SM1005
SM1008	Δ*barA*::*kan*/p-*barA*	SM1006
SM1009	Δ*barA*::*kan* Δ*uvrY*::*cam*	SM1006
SM1010	Δ*uvrY*::*cm*/p-*uvrY*	SM1007
SM1020	Δ*cyaA*::*kan*	SM1005
SM1021	Δ*uvrY*::*cam* Δ*cyaA*::*kan*	SM1020
SM1030	Δ*csrA*::*kan*	SM1005
SM1031	Δ*csrA*::*kan*/p-*csrA*	SM1030
SM1040	Δ*crp*::*kan*	SM1005
SM1041	Δ*uvrY*::*cam* Δ*crp*::*kan*	SM1007
SM1050	Δ*hfq*::*cam*	This study
SM1051	Δ*hfq*::*cam*/p*hfq*	SM1050
SM1060	MG1655 Δ*lac* Δ*uvrY*::*cam*/pLW11	SM1002
SM1061	MG1655 Δ*lac* Δ*csrA*::*kan/*pLW11	SM1003
BB170	*luxN*::Tn5	[43]
BB152	*luxL*::Tn5	[43]
pBR322	Cloning vector	Lab
pCR2.1	Cloning vector	Invitrogen
pKD46	For arabinose induction of λ Red System	[38]
pKD3 and pKD4	Contains *kan* and *cat* gene respectively for λ Red knockout	[38]
pLW11	Contains *lsrACDBFG* promoter, Amp^r^	[62]
pSP417	Modified pRS415 for cloning *lacZ* transcriptional fusion, Amp^R^	[40]
pCA132	The *csrA* gene in pFF584 with pSC101 *ori*	[42]
pSM2	*uvrY* within the EcoRV-BamHI site of pBR322, Amp^R^	This study
pSM3	*luxS* gene in the EcoRI site of pBR322, Amp^R^	This study
pSM4	469 bp 5’ of *luxS* within SalI-SmaI site of pSP417	This study

### Recombinant DNA techniques

Standard molecular techniques were used for cloning [37]. Amplifications for cloning were performed by Tgo polymerase and other amplifications by Taq or Pfx polymerase. PCR products were cloned into pCR2.1 using the TOPO-TA cloning system (Invitrogen, Carlsbad, CA) and few clones were verified by sequence analysis. The *uvrY* gene was cloned in pBR322 generating pSM2 (p-*uvrY*) as described earlier [31]. Similarly the *luxS* gene was amplified using OSM34 and OSM35 (Table 2) and cloned into pCR2.1. A 700 bp EcoRI fragment was subsequently cloned into EcoRI site of pBR322 creating pSM3 (p-*luxS*). Both the *luxS* and *uvrY* open reading frame were oriented in the same direction as the *tet* gene in the vector.

**Table 2:** List of oligonucleotides used in the study.

Primer Name	Primer purpose	Sequence (5’-3’)
OSM34	luxS–F	GTGAAGCTTGTTTACTGACTAGAT
OSM35	luxS–R	GTGTCTAGAAAAACACGCCTGACAG
OSM43	uvrY KO—F	TGGTGCCGCCAGGGATACGACGCATTCTGGAAGTTGCATATGAATTCCTCCTTAGT
OSM44	uvrY KO—R	CATTTGTTGAGCGATGTCAGAAGCAATGTAACGCTGACCGTGTAGGCTGGAGCTGCTTC
OSM49	luxS KO–FWD	TGCGCTTCTGCGTGCCGAACAAAGAAGTGATGCCAGTTGCATATGAATATCCTCCTTAGT
OSM50	luxS KO-REV	CACGCTGCTCATCTGGCTGTACCAATCAGACTCATATACTGTGTAGGCTGGAGCTGCTTCG
OSM53	luxS–F	CCCGTCGACATAGCATTTGCAGAAGCCTACCGTA
OSM54	luxS–R	CCCGGGCCCATACAAACAGGTGCTCCAGGGTATG
OSM55	T7 –luxS (F)	TAATACGACTCACTATA**G**GGAGA*GGCTGGAAAAACAC*
OSM56	T7 –luxS (R)	CGCTTCCATCCGGGTATGATCG
OSM59	luxS-FWD	TGATCCTGCACTTTCAGCAC
OSM60	luxS-RV	CAATCACCGTGTTCGATCTG
OSM61	rrnA-F	AGCGTTCTGTAAGCCTGTGAAGGT
OSM62	rrnA-R	TAACGTTGGACAGGAACCCTTGGT
OSM63	icd-F	GGAATCGGTGTAGATGTAACCCC
OSM64	icd-R	CGTCCTGACCATAAACCTGTGTGG
OSM 70	ChIP–csrA	CACGGTGACCTCATCCCCAATC
OSM71	ChIP–csrA	TACGGATGCTGCGGCCTTACCTG
OSM72	csrB promoter	CCTGCGTAAATCGGAGTTTAGAAC
OSM72	csrB promter	GTGTGGTGGGGCTACACTATGAAG
OSM 75	lsrK–F	GGCACATTCTGGCAGCAAGTTGTA
OSM76	lsrK-R	TTTCTTCGGCACAGAAAGCATCGC
OSM 77	lsrA-F	TGCGCCCTTACTCATAACCTTCGT
OSM 78	lsrA-R	CAATACTTGCGGCGAAGCTTCCAA
OSM 79	lsrR-F	AACCACAACAGATGCTGGCGATTG
OSM 80	lsrR-R	TTAAGCTGCCCGATTCCCGTCATA

### Construction of chromosomal deletion insertion mutants

The *uvrY* and *luxS* genes in MG1655 were disrupted by lambda red recombination method [38]. The *uvrY* gene was deleted and replaced with a chloramphenicol cassette by using the primers OSM43 and OSM44. The *luxS* gene was similarly deleted with a kanamycin cassette by using the primers OSM49 and OSM50. P1*vir* transductions were performed as earlier described [39]. Mutations were transduced into relevant background whenever necessary and characterized for known phenotypes.

### Construction of chromosomal *luxS-lacZ* transcriptional fusion

Since a disruption of the *luxS* gene causes growth-defect, we constructed a merodiploid strain with a single-copy *luxS-lacZ* transcriptional fusion incorporating upstream sequence from the *luxS* ATG codon. A 469 bp fragment incorporating 290 bp upstream regulatory sequences region and 59 codons of *luxS* gene were PCR amplified with Tgo polymerase from MG1655 chromosomal DNA using primers OSM53 which includes a SalI restriction site and OSM54 which includes a SmaI restriction site. The amplified fragment was cloned within the SalI-SmaI site of promoterless *lacZ* transcriptional fusion vector pSP417, a modified pRS415 vector with extended multiple cloning sites [40, 41]. The clones were sequenced to check the integrity of the amplified fragment and the fusion junction. The plasmid-borne fusion was transferred to λRS45. The resulting recombinant phage, λP*luxS-lacZ* (λSM001) was used to transfer the fusion into MG1655Δ*lac*, creating a merodiploid *luxS*^+^ *luxS-lacZ* fusion (SM105). Single-copy fusions were isolated and verified by a Ter assay followed by measuring β-galactosidase activity. A single copy fusion integrated within the λ *att* site of the *E*. *coli* chromosome was selected to study *luxS* expression under various experimental conditions. We also observed that when a *csrA*::*kan* mutation was transduced from the parent strain TR1-5 MG1655, the resultant phenotype was that of a very slow growing strain as reported in *S*. *typhimurium* [42]. Because normal growing suppressors could be easily isolated after prolonged growth with aeration in LB broth, we selected single copy λΦ(*luxS’-‘lacZ*) (hereafter referred as *luxS-lacZ*) fusion in TR1-5 MG1655.

### Enzymatic assays

The extracellular AI-2 in cell-free supernatant was assayed using *V*. *harveyi* strain BB170 as described [43]. The reporter strain BB170, a *luxN* mutant of BB120 was chosen because of its sensitivity to AI-2 but not to AI-1. The positive controls were either BB152 (AI-1^-^, AI-2^+^) or BB120 (AI-1^+^ AI-2^+^) and the negative control was *Escherichia coli* DH5α, a *luxS* mutant which was unable to synthesize AI-2. *V*. *harveyi* was cultured in autoinducer bioassay (AB) medium The *V*. *harveyi* reporter strains were grown overnight (~16 h) at 30°C on rotating wheels in AB medium, diluted 1:2500 into fresh medium and 180 μl of the diluted cells were added to microtiter wells (Nalge Nunc, Rochester, NY) alongwith 20 μl of the cell-free culture supernatants. To minimize fluctuations in luminescence and light scattering a higher volume per well was used. The microtiter plates were incubated at 30°C on a rotary shaker at 150 rp m. Light production was measured every 30 minutes using a Mediators PhL™ Luminometer (ImmTech, Inc, New Windsor, MD) or by a VICTOR^3^™V Multilabel Counter (PerkinElmer) for 24 hours. Serial dilutions of *V*. *harveyi* BB120 (wild type) and DH5α 13h-old culture supernatant were used as a positive and negative control respectively. The relative AI-2 activity was reported in relative light units (RLU) where background reading of media and surface is subtracted from the actual reading as directly reported by the instrument for each plate. β-galactosidase assay was determined as described [39]. All assays were performed in duplicate and repeated three times.

### Electrophoretic Mobility Shift Assay

Since many response regulators can be phosphorylated by acetyl phosphate, we wanted to determine whether UvrY requires phosphorylation in order to interact with *luxS* promoter. The *luxS* promoter DNA was radiolabeled and various concentration of purified UvrY protein ranging from 1 to 2.5μM was used for gel-shift analysis. Purified UvrY was phosphorylated with 20 mM acetyl phosphate. Cold DNA was added to determine the strength of the interaction of the protein with the promoter.

### Chromatin Immunoprecipitation assay

In vitro binding of UvrY protein to various promoters was determined by methods described previously [44, 45]. Briefly, cultures were grown to an O. D. _600_ of 0.8 and the expression His6-UvrY was induced for 1 h with 1 mM IPTG at 37°C under aerobic conditions. Formaldehyde was added at 1% final concentration to the growing cultures. After 20 min, 0.5 M glycine was added to quench the reaction, and the culture was placed on ice for another 20 minutes. The cells were subsequently washed twice with PBS (pH 7.5) and re-suspended in 1/100^th^ volume of lysis buffer (50 mM Tris HCl, pH 7.4, 150 mM NaCl, 1mM EDTA, 1% Triton X-100) with lysozyme and PMSF added to final concentrations of 4 mg/ml and 1 mM respectively. After 15 minutes of incubation at 37°C, cells were lysed and DNA sheared by sonicating in a cup horn sonicator (Misonix 3000, Farmingdale, NY). After removal of cell debris, the clear lysate was used for immunoprecipitation using anti-UvrY antibody linked to agarose beads. 300 μl of clear supernatant was added to 80 μl of antibody conjugated resin and mixed at room temperature with shaking for 2 h. The mixture was washed three times with 1 ml wash buffer (50 mM Tris HCl, pH 7.4, 150 mM NaCl). 100 μl of elution buffer (0.1 M Glycine, pH 3.5) with 1% SDS was used to elute the antibody-captured proteins from the resin. The clear supernatant comprising of approximately 500 bp DNA fragments, was further de-crosslinked and de-proteinized overnight at 65°C in the wash buffer containing1 mg/ml of Proteinase K. 50 μl of the de-crosslinked reaction was then cleaned using QIAquick PCR purification (Qiagen, Valencia, CA) and subsequently used to test presence of target promoters. The ChIP assay was repeated with a Flag-UvrY and anti-Flag antibody (Sigma, St. Louis, MO), confirming similar results.

### Real time RT-PCR

Total RNA was isolated from wild type and relevant mutants at early exponential phase, mid-exponential phase and stationary phases and then subjected to qRT-PCR analysis with *rrnA* transcript as an internal control as described earlier [31]. Quantitative polymerase chain reaction (qPCR) and quantitative real-time polymerase chain reaction (qRT-PCR) were performed as per the manufacturer’s recommendations. For qPCR, the first-strand cDNA was synthesized from 5μg of total RNA using Moloney Murine Leukemia Virus Reverse Transcriptase, Superscript II RNase H^-^ (Invitrogen, Carlsbad, CA) and 50 ng of random hexamers (Invitrogen, Carlsbad, CA). For the PCR reaction, 10 ng of first-strand cDNA was amplified separately with 10 μM each of gene-specific primer and 16S *rrnA* gene-specific primers in a 25 μl total reaction volume with Taq polymerase in a Biometra T-Gradient PCR instrument (Biometra, Horsham, PA) for 30 cycles. At the end of several cycles, a gene-specific and an *rrnA*-specific reaction tube was removed. Five μl of the reaction products were resolved separately in a 1.2% agarose gel and the product intensities were quantitated by a BioRad Gel Documentation system (BioRad, Hercules, CA). The linear range of amplification for the *rrnA* gene was from 5–15 cycles in all backgrounds, while that of the *luxS* were from 12–22 cycles in the wild-type strain, and appeared later in the mutants. A qRT-PCR reaction was performed on the above set of samples under identical reaction conditions in a LightCycler (Roche, Indianapolis, IN) with SYBR Green-1 PCR Master Mix. The fluorescence signal from SYBR Green intercalation was monitored to quantify double-stranded DNA product formed after each PCR cycle. The threshold cycle for which a statistically significant increase in the amount of the PCR product is detected is denoted as Ct. Starting with individual cDNA pools from various genetic backgrounds, Ct values were determined for *rrnA* and *luxS* amplification products. The ΔCt values between samples derived from various strains were normalized with the *rrnA* product, as ΔCt = − (Ct_*rrnA*_ − Ct_wild-type_) − (Ct_*rrnA*_ − Ct_mutant_). Since PCR products double with each amplification cycle, the fold difference in the initial concentration of each transcript is determined by 2^ΔCt^. The results derived from gel-based experiment indicated slightly less difference than the SYBR Green fluorescent method.

### Rifampicin chase assay

RNA stability assay was performed as described earlier [31]. Briefly total RNA was isolated from cells at the entry of stationary phase when *csrA* is maximally expressed. Rifampicin was added to inhibit transcription initiation and before and after 2.5, 7.5 and 10 minutes post addition of rifampicin, total RNA was isolated and RT-PCR was performed. Rifmapicin was added at a final concentration of 500 μg/ml. A housekeeping control *icd* was kept as the internal control. Gene specific primers were used to amplify *luxS* and *icd* message (Table 2).

## Results

### Effect of extracellular AI-2 accumulation in *uvrY* mutant

**Fig 1:**
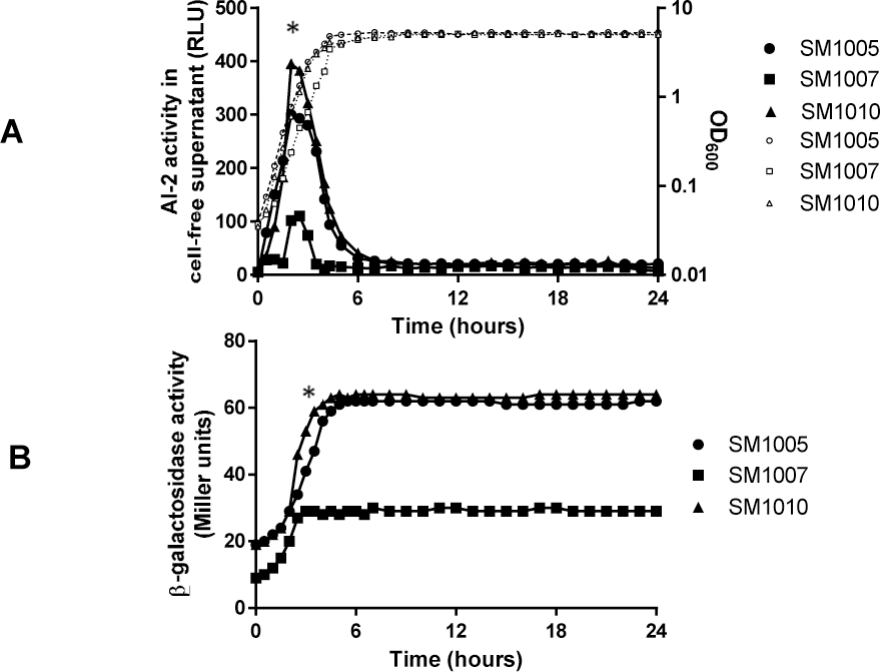
Effect of *uvrY* on AI-2 accumulation and expression of *luxS*::*lacZ* fusion. (A) AI-2 accumulation in the supernatant was assayed by *Vibrio harveyi* reporter assay in SM1005 (*luxS*::*lacZ*), SM1007 (Δ*uvrY luxS*::*lacZ*) and SM1010 (Δ*uvrY/*p*uvrY luxS*::*lacZ*) for a period of 24 hours. (B) Expression of the fusion in the strains was monitored by β-galactosidase assay for 24 hours. Dotted lines indicate growth of corresponding bacterial strains. The graphs represent mean of three experiments. Asterisk marks are provided for a representative data point used for calculation of significance.

The AI-2 accumulation of *E*. *coli* grown in LB broth was growth phase-dependent in consistence with previous studies [16, 46]. The accumulation of AI-2 in the extracellular milieu peaks at the mid logarithmic phase, declines at the entry of stationary phase and vanishes once the cells shift deep within stationary phase of the growth cycle. No secondary peak of AI-2 was observed within a span of 24 hours. A mutation in the *uvrY* gene reduced AI-2 accumulation in the extracellular milieu compared to the isogenic wild-type (Fig 1A). The difference was pronounced in mid-exponential and in early stationary phase. In the complemented strain, the extracellular AI-2 accumulation was similar to the wild type. A very low level of AI-2 was detected in the *luxS*::*kan* mutant from cell free supernatant, and plasmid complementation in the mutant increased AI-2 levels to that of the wild type (S1 Fig).

### Expression of *luxS-lacZ* transcriptional fusion in the *uvrY* mutant

The growth dependent AI-2 expression correlates with *luxS* expression until the culture reaches the stationary phase (Fig 1B). The expression of the *luxS-lacZ* fusion in the *uvrY*::*cam* mutant was significantly lower in mid-exponential and early stationary phase as compared to the wild type. The basal level of expression of the fusion in *uvrY*::*cam* mutant was around 2-fold lower than wild type in both mid-exponential and stationary-phase and plasmid complementation of *uvrY* in the mutant partly restores the wild type expression levels (Fig 1B).

### Mutation in *uvrY* reduced expression of *luxS* transcript

Transcription of *luxS* was evaluated by quantitative reverse transcription PCR analysis and Northern analysis (Table 3 and S2 Fig). On qRT-PCR, we observed visible *rrnA* amplification products by 9^th^ cycle, and the *luxS* transcript in the 12^th^ cycle in the wild-type strain. Although the accumulation of *rrnA* product remained consistent for the mutant strains, visible *luxS* amplification was observed around 16^th^ cycles for *uvrY*::*cm* and in the double mutant strain. The experiment was repeated using a more sensitive Real Time Light Cycler (Roche, Indianapolis, IN) using SYBR-Green to follow the simultaneous amplification of the *rrnA* product with that of the *luxS* products from the same initial cDNA sample. Northern blotting was further performed to quantify the level of *luxS* transcript. Loss of *uvrY* resulted in reduced expression of *luxS* transcript (~3.3 fold) which could be restored upon plasmid complementation (S2 Fig).

**Table 3:** The BarA-UvrY TCS regulates *luxS* transcription as determined by qRT-PCR.

Strain	Relevant Genotype	C_t_ Values ^a^		Fold difference ^b^ (2^ΔCt^)
		*rrnA*	*luxS*	
MG1655Δ*lac*	Wild type	6.5 ± (0.5)^c^	23.5 ± (0.6)	1.0
SM1006	Δ*barA*::*kan*	6.5 ± (0.5)	26.0 ± (0.2)	5.6 / 5.6 ^d^
SM1007	Δ*uvrY*::*cm*	6.5 ± (0.5)	25.5 ± (0.3)	4.0 / 4.7
SM1009	Δ*barA* Δ*uvrY*	6.5 ± (0.5)	25.5 ± (0.3)	4.0 / 2.8

^a^ Ct values are the threshold values of PCR cycles where the SYBR Green fluorescence was detected above background in the linear range, taken at 7.0 Relative Light Units.

^b^ The fold down-regulation is calculated as 2^ΔCt^, Where ΔC_t_ = (C_t *wt*_—C_t *rrnA*_)–(C_t mutant_—C_t *rrnA*_).

^c^ Standard deviation of three independent experiments.

^d^ Fold-difference of the *luxS* transcript transcript normalized with *rrnA* levels and compared to the wild-type strain.

### *In vitro* and *in vivo* interaction of UvrY with *luxS* promoter

**Fig 2:**
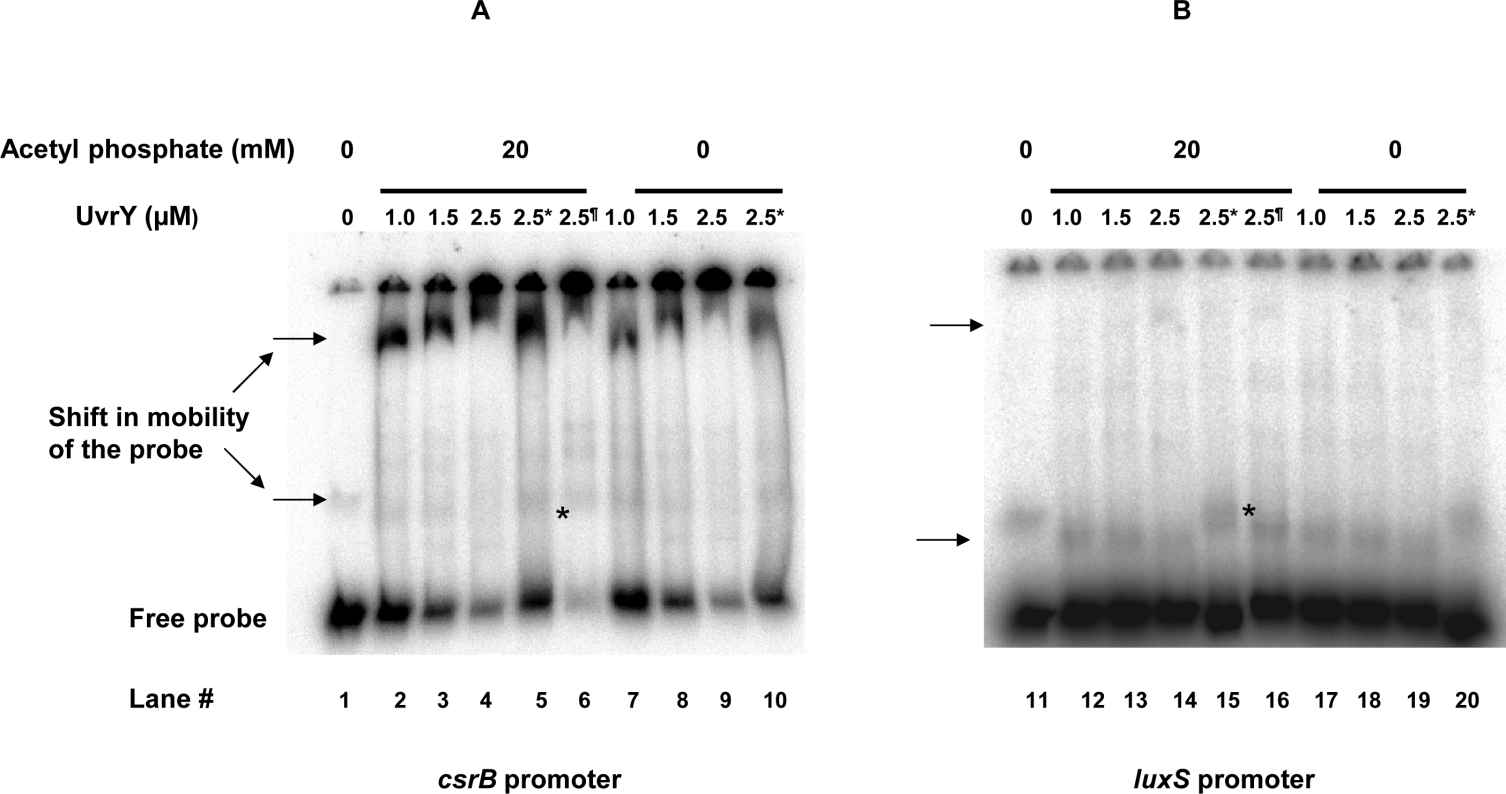
**Electrophoretic mobility shift assay (EMSA) demonstrating interaction of UvrY with A) labeled *csrB* B) labeled *luxS* promoter.** The promoter DNA was radiolabelled with P^32^. Purified UvrY was added at indicated concentrations without and with 20mM acetyl phosphate. The shift in probe was indicated by an arrow. Unlabeled DNA were added as negative and positive competitors shown by * and ¶ respectively.

**Fig 3:**
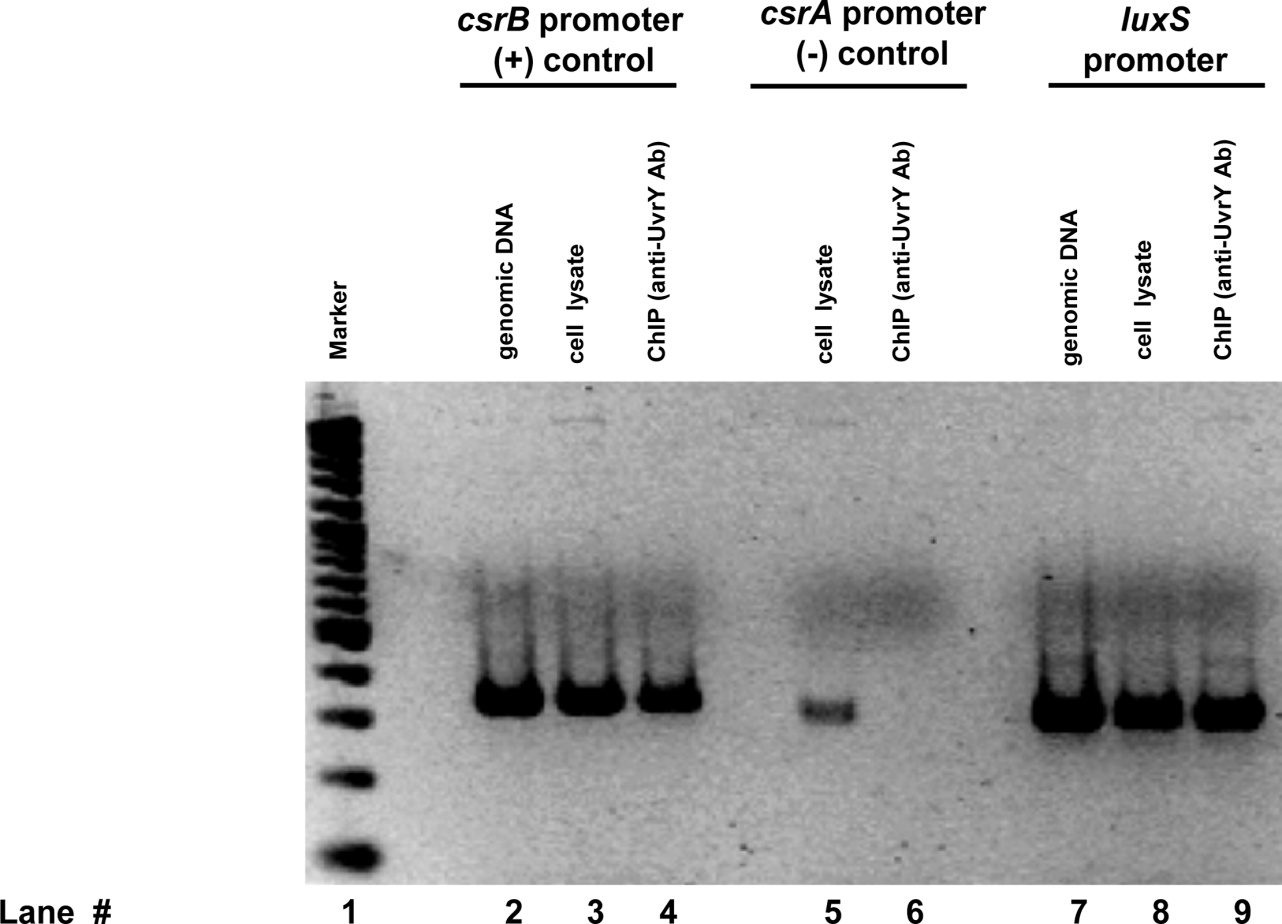
Direct *in-vivo* binding of UvrY with *luxS* promoter by chromatin immunoprecipitation assay (ChIP). The occupancy of UvrY at the *csrA*, *csrB* and *luxS* promoters in *E*. *coli* was analyzed by ChIP using an anti-UvrY antibody. Purified DNA was PCR amplified using target specific primers. Genomic DNA and cell lysates were used as template for additional controls in PCR reaction. A 100 bp ladder marker was used for size comparison of amplicons. Expected sizes for all amplicons were between 300–400 bp.

Purified UvrY interacts with *luxS* promoter without and with acetyl phosphate (Fig 2). The shift in the probe was shown by an arrow. The interaction of UvrY with *luxS* promoter is relatively weak compared to that with *csrB* promoter. Furthermore, interaction was also modulated by addition of negative and positive competitor DNA. Addition of positive competitor releases the binding whereas that of negative competitor tightens it. Since the interaction of *uvrY* with *luxS* promoter was relatively weak indicating there may be yet other factors associated with the interaction, we performed a in vivo chromatin immunoprecipitation of the *luxS* promoter fragment to determine the *in vivo* interaction using anti-uvrY antibody. Our result confirms the in-vivo binding of UvrY with *luxS* promoter (Fig 3). For the control reactions, we found the *in vivo* interaction of UvrY with *csrB* is relatively strong, while that with *csrA* was very weak, as expected from previous studies.

### UvrY is required for cAMP-CRP repression of *luxS*-lacZ expression

**Fig 4:**
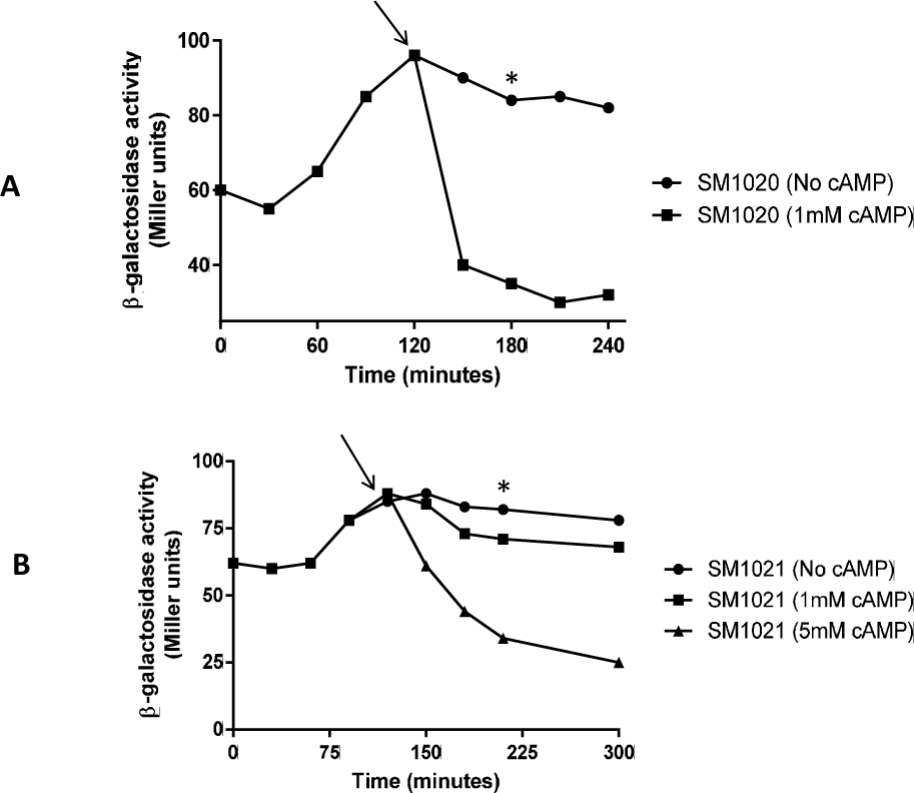
UvrY influences cAMP-CRP catabolite repression of *luxS*. A) Expression of *luxS*::*lacZ* in SM1020 (Δ*cyaA*::*kan luxS*::*lacZ*) in the absence of cAMP and in the presence of 1 mM cAMP. (B) Expression of *luxS*::*lacZ* in SM1021 (Δ*uvrY*::*cam* Δ*cyaA*::*kan luxS*::*lacZ*) in the absence of cAMP, 1mM cAMP, and 5 mM cAMP. The point of cAMP addition is marked with an arrow on the graphs. Mean of three experiments were plotted and a representative data point used for calculation of significance is shown in asterisk (*p*<0.05).

**Fig 5:**
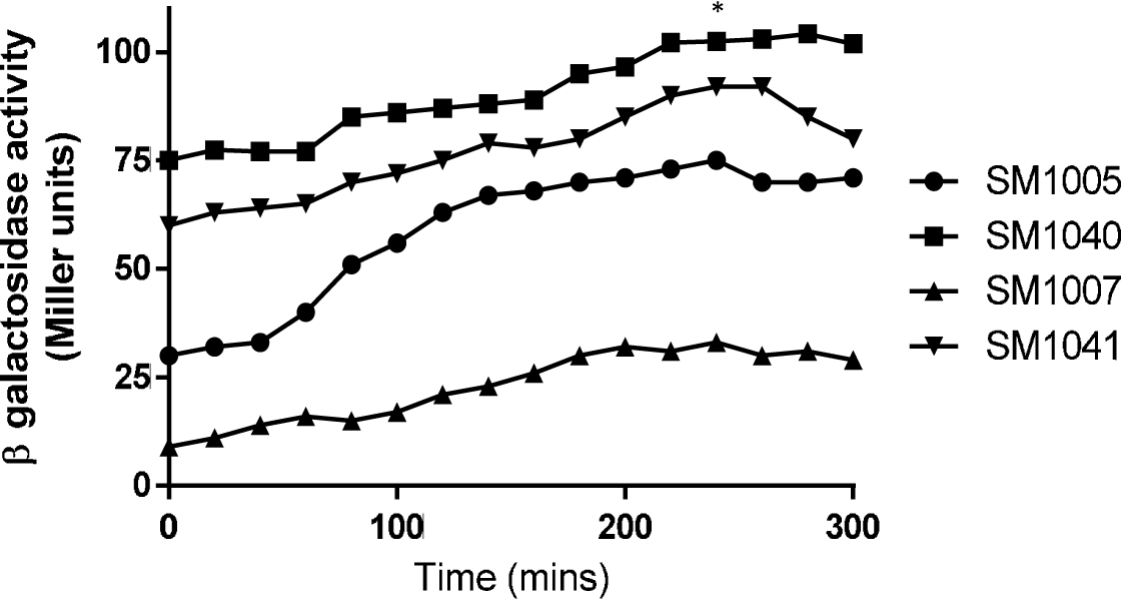
Effect of *crp* on *luxS*::*lacZ* expression in absence or presence of *uvrY*. Strains SM1005 (*luxS*::*lacZ*), SM1007 (Δ*uvrY luxS*::*lacZ*), SM1040 (Δ*crp*::*kan luxS*::*lacZ*) and SM1041 (Δ*crp*::*kan* Δ*uvrY*::*cam luxS*::*lacZ*) were grown in TB and β-galactosidase expression was measured at indicated time points. This experiment was repeated three times and mean was plotted in the graph.

We further wanted to test any additional regulator might be involved in expression of *luxS-lacZ* fusion. An important regulation occurs through the cyclic AMP-cyclic AMP Receptor Protein (cAMP-CRP) system. To further determine the role of UvrY in the glucose repression of *luxS-lacZ* fusion, we deleted adenylate cyclase encoding gene *cyaA*, which blockssynthesis of cAMP from ATP. Disruption of *cya* gene led to a constitutive expression of the *luxS-lacZ* fusion (Fig 4A). Addition of 1mM cAMP reduced the expression of *luxS* fusion to a basal level in the mutant. In case of Δ*uvrY*Δ*cya* double mutant, the expression of the fusion is constitutive and addition of 1mM cAMP reduced the expression marginally (Fig 4B). The repression was faster in a Δ*cya* mutant (30 min) compared to a Δ*uvrY*Δ*cya* mutant (~90 min). Only addition of 5mM reduced the expression of the double mutant to a basal level. Similarly, deletion of *crp* resulted in constitutive expression of *luxS* and a Δ*uvrY*Δ*crp* mutant reduced the expression of the fusion marginally (Fig 5).

### Effect of extracellular AI-2 accumulation and expression of *luxS-lacZ* fusion in *csrA* mutant

**Fig 6:**
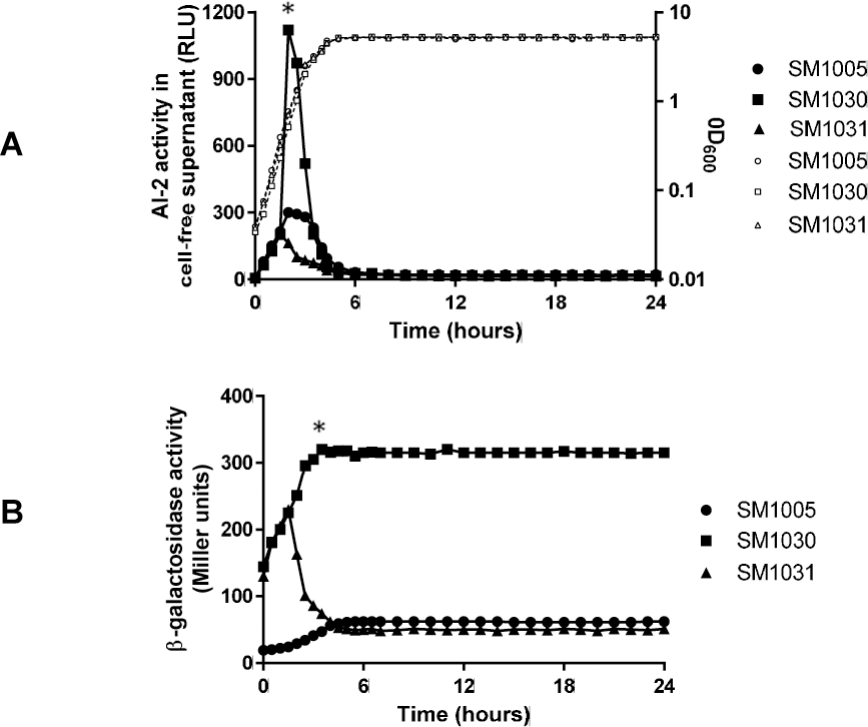
Extracellular accumulation of AI-2 and expression of *luxS*::*lacZ* fusion in a *csrA* mutant. (A) AI-2 accumulation in the supernatant was assayed by *Vibrio harveyi* reporter assay in SM1005 (*luxS*::*lacZ*), SM1030 (Δ*csrA luxS*::*lacZ*) and SM1031 (Δ*csrA*/p*csrA luxS*::*lacZ*) (B) Expression of *luxS*::*lacZ* was monitored by β-galactosidase assay in same sets of strains at indicted time points. Dotted lines indicate growth of corresponding bacterial strains. The graphs represent mean of three experiments. Representative data point used for calculation of significance is shown in asterisk.

In contrast to *uvrY* mutant, mutation in the *csrA* leads to elevated levels of AI-2 relative to the wild type and complementation of *csrA* in the mutant reduced extracellular accumulation of AI-2 to the wild-type level (Fig 6A). The inverse correlation of AI-2 accumulation in the *uvrY* and *csrA* deficient strains suggested a regulation both at the level of transcription and post transcription of *luxS* and AI-2 uptake. The uptake of AI-2 was also evaluated in the *uvrY* and *csrA* mutants and indicated an inverse relationship in the *lsr* operon expression. Similarly in case of the *csrA* mutant, the expression of the fusion was significantly higher at the entry of stationary phase as compared to the wild type while complementation of the mutant restored the expression to the wild type level (Fig 6B). Interestingly, the expression pattern was similar to that of the wild-type strain, with maximum expression being observed as cells enter the stationary phase. A mutation in the *csrB* gene resulted in marginally higher expression of the *luxS-lacZ* fusion.

### Regulatory interaction of CsrA with *luxS* mRNA stability or *luxS* mRNA leader sequence

**Fig 7:**
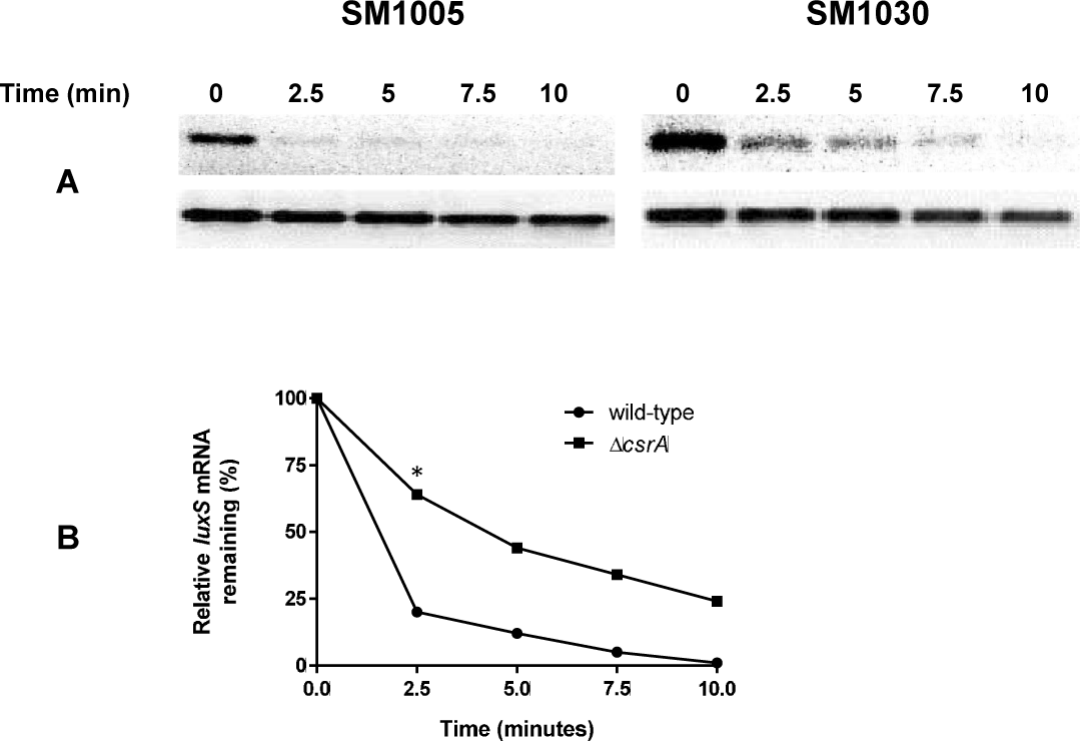
Effect of *csrA* on message stability of *luxS* in *E*. *coli*. Total RNA was isolated from late logarithmic growth phase. (A) Message stability of *luxS* or *icd* (housekeeping control) was evaluated before and following addition of rifampicin for 10 minutes. (B) Relative intensity of the *luxS* in the wild-type and the mutant as compared to intensity of *icd* before addition of rifampicin was set at 100%. This experiment was repeated two times.

**Fig 8:**
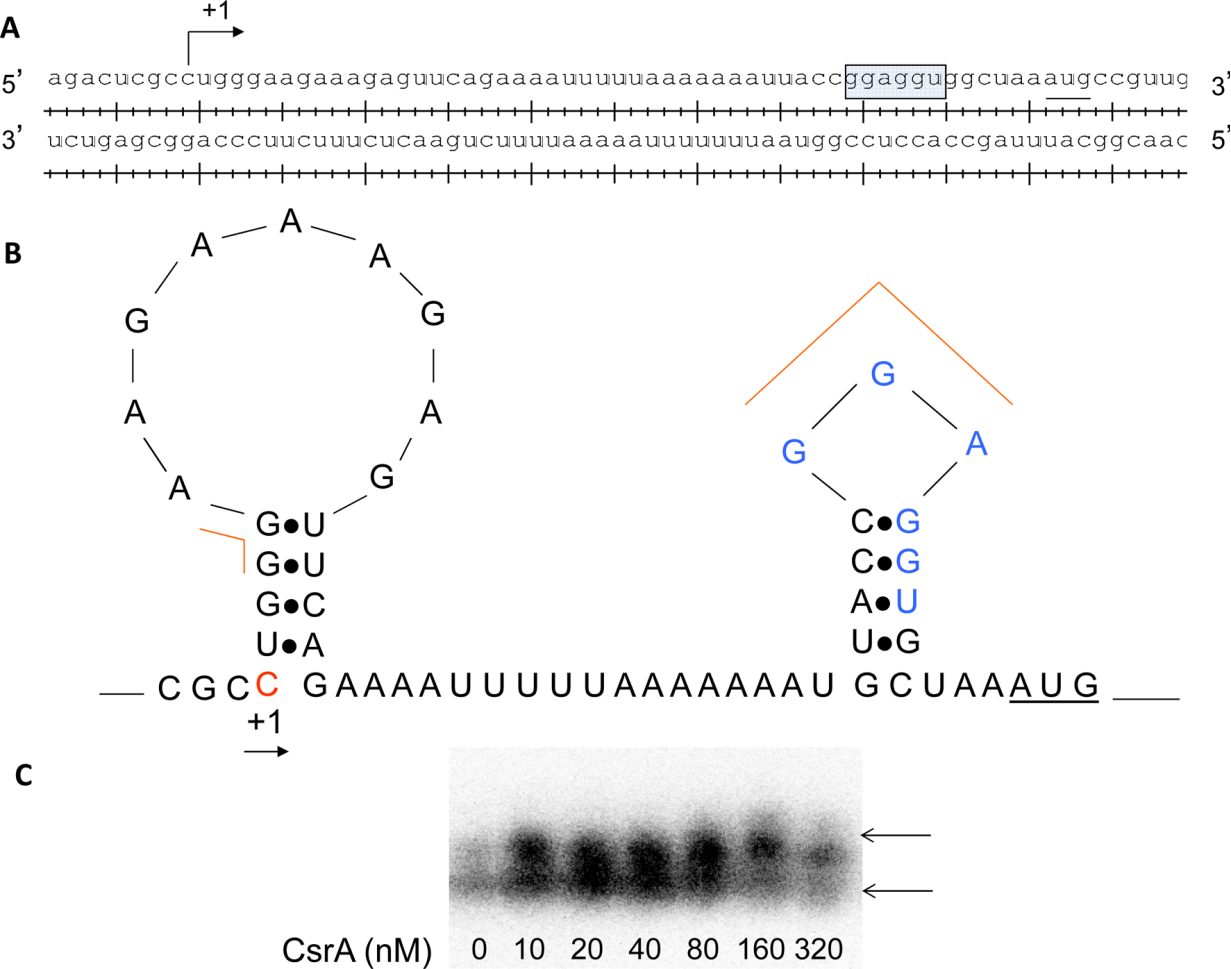
Regulatory interactions of CsrA with *luxS* upstream region. A. Transcription start site and ribosome binding sites of *luxS* were indicated. B. Predicted binding sites of CsrA in *luxS* upstream regulatory region. C. CsrA-luxS mRNA regulatory interactions. 5’- end labeled *luxS* leader transcript was incubated with CsrA at concentration as shown below each lane. Positions of free and bound RNA are shown. The shift in the labeled probe was shown by an arrow.

We evaluated the effect of *csrA* in *luxS* transcript stability. At the entry into stationary phase, loss of *csrA* resulted in stabilization of *luxS* transcript relative to the wild-type straining harboring *csrA* (Fig 7A). Quantification of the DNA intensities by ImageJ suggested a three-fold reduction in the half-life of *luxS* transcript in the wild-type relative to the Δ*csrA* mutant (Fig 7B). CsrA is known to bind leader regions of target genes with high affinity for GGA sites. We predicted the potential binding sites for CsrA in the *luxS* leader region with GGA sites using Lasergene program suite (DNAstar, Madison, WI, USA), (Fig 8A). Folding of the leader RNA using RNAFold in the Vienna RNA website indicated that one of the GGA site is actually present within the ribosome binding site of *luxS* (Fig 8B). Our results indicate that CsrA stabilizes *luxS* message stability at the entry into stationary phase. We also determined the interaction of CsrA with *luxS* leader region. Our results indicate that CsrA binds to *luxS* leader region and influences *luxS* message stability (Fig 8C). We also determined the effect of *hfq* on the *luxS* fusion expression. We found that loss of *hfq* reduced the *luxS* fusion expression significantly and complementation of *hfq* in the mutant restored *luxS* expression to wild-type levels (S3 Fig).

### Effect of expression of Lsr promoter,*lsrA* and *lsrk* in *uvrY* and *csrA* mutant

**Fig 9:**
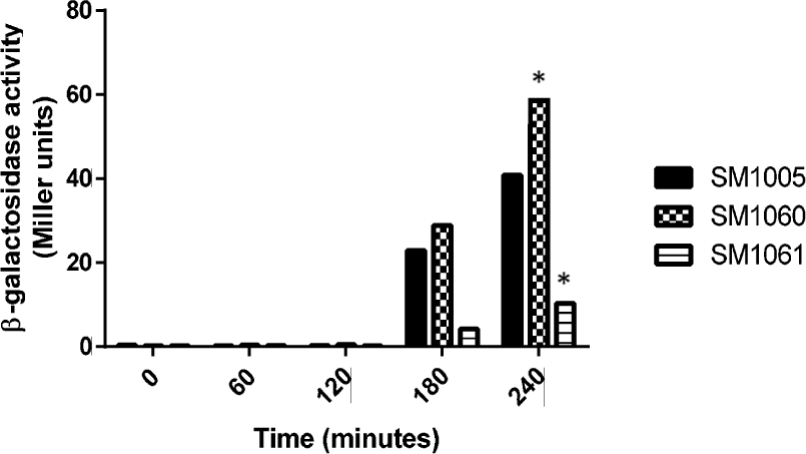
Effect of *lsr* promoter activity in *uvrY* and *csrA* mutant. SM1059 (wild type/p-lsr), SM1060 (Δ*uvrY*/p-lsr) and SM1061 (Δ*csrA*/p-lsr) were tested for the expression of Lsr promoter activity. This experiment was repeated three times and mean of three experiments for each time points were plotted (*p*<0.05). Representative data point used for calculation of significance is shown in asterisk.

In order to determine whether *uvrY* and *csrA* play a role in the regulation of uptake of AI-2 signal, the expression of the *lsrA* or *lsrK* or the Lsr promoter was evaluated in *uvrY* and *csrA* mutant. The Lsr promoter activity increased marginally in Δ*uvrY* mutant and approximately four-fold reduced in the *csrA* mutant at the entry of stationary phase (Fig 9). Expression of the *lsrR* was reduced both in the *uvrY* and *csrA* mutant (Table 4). The uptake of AI-2 was also evaluated in the *uvrY* and *csrA* mutants and indicated an inverse relationship in the *lsr* operon expression.

**Table 4:** Effect of mutation of *uvrY* and *csrA* on expression of Lsr transporter.

Relevant Genotype	Relative mRNA level			β-galactosidase (Miller Units) P*lsr*::*lacZ*
	*lsrK*	*lsrR*	*lsrA*	
Wild-type	100	100	100	40.8 ± 3.5
*ΔuvrY*::*cam*	83.0 ± 1.5	34.0 ± 1.5	90 ± 1.0	58.6 ± 3.5
*ΔcsrA*::*kan*	82.0 ± 1.3	45 ± 1.5	44 + 1.5	10.3 ± 1.5

### Predicted integrative model of AI-2 based cell-signaling and the BarA/UvrY/CsrA pathway

**Fig 10:**
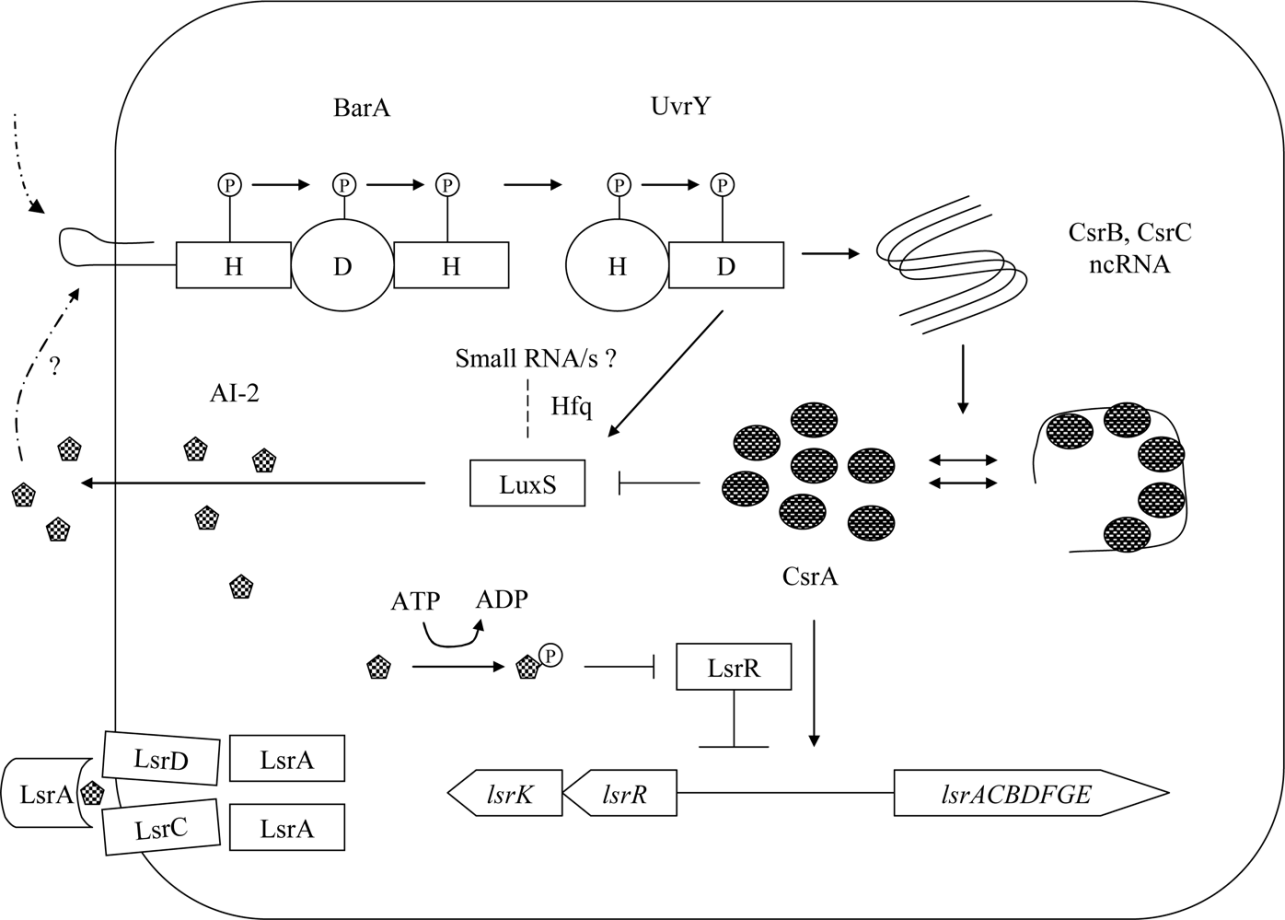
Regulatory circuit of the BarA/UvrY/CsrA with AI-2 based cell-to-cell signaling. The BarA/UvrY TCS regulates *luxS* expression positively at transcriptional level whereas CsrA negatively regulates *luxS* post-transcriptionally. A possible role of quorum sensing regulatory RNA is indicated in the circuit.

We predicted a model integrating the BarA/UvrY/CsrA pathway with AI-2 based signaling (Fig 10). The BarA/UvrY/CsrA pathway controls both the synthesis and uptake of AI-2 via transcriptional and posttranscriptional mode of regulation of *luxS* and *lsr* transporter. In this model both BarA, the sensor kinase and UvrY, the response regulator positively regulates the expression of *luxS* and AI-2 accumulation. CsrA on the other hand, negatively regulates *luxS* expression and AI-2 accumulation in the extracellular milieu. By contrast, the BarA/UvrY negatively controls Lsr promoter activity, whereas CsrA positively controls Lsr promoter activity. Such regulation suggests a balance between synthesis and uptake of AI-2 in *E*. *coli*. The repression of *luxS* is dependent on cAMP-CRP and *uvrY* influences this regulation. The role of small RNAs could be important for regulation of *luxS* in *E*. *coli*.

## Discussions

Quorum sensing is a bacterial population dependent cell-to-cell signaling mechanism mediated via synthesis, release and uptake of small freely diffusible molecules called autoinducers [47, 48]. Apart from the requirement of QS on cell-density, other environmental cues such as pH, nutrient depletion and stress are important for quorum sensing systems to synchronize coordinated response for adaptation and survival of bacteria [49]. In *Pseudomonas aeruginosa*, the integration of metabolic cues with cell-cell signaling requires several regulators at the level of transcription and post transcription [50]. Metabolic adaptation through two-component regulatory system is achieved by signal detection and response by appropriate changes in gene expression by the cognate response regulator.

The conserved pathways of BarA/UvrY/CsrA and its orthologs in the γ-subdivision of proteobacteria regulates virulence and secondary metabolism in *E*. *coli*, *S*. *typhimurium*, *P*. *fluorescens* and *V*. *fischeri* [51–58]. Our earlier work in *E*. *coli* demonstrated the pleiotropic role of BarA/UvrY/CsrA pathway in regulation of biofilm formation, motility and virulence gene expression [29–31]. Here we have shown the association of this regulatory pathway with *luxS* based AI-2 quorum sensing. Our data shows that multiple regulatory factors control the expression of LuxS including the BarA/UvrY/CsrA pathway, and global virulence regulator, *crp* and small regulatory RNAs. We found the interaction of UvrY with *luxS* promoter is very weak *in vitro* and that the interaction can be demonstrated *in vivo*. This suggests that there are other additional factors such as DNA bending proteins (HU, IHF, etc.) that might play a role in this regulation [59]. These findings indicate that the BarA/UvrY/CsrA pathway integrates with AI-2 based cell-cell signaling in *Escherichia coli* and as a consequence the regulatory pathway play a critical role in metabolism, persistence, virulence and other community associated microbial behaviors. The control of synthesis and uptake of AI-2 suggest regulatory crosstalk of metabolic pathways with cell-cell signaling. Our results are similar to the regulation in *Vibrio cholera* where CsrA and three small RNA’s regulate quorum sensing [60]. VarS/VarA two-component regulatory system in *Vibrio cholera* also regulates quorum sensing [20]. Furthermore *luxS* influences biofilm formation in both AI-2 and AI-2 independent manner in *E*. *coli* [61]. Our results further are in consistence with the role of cAMP-CRP mediated repression of *luxS* in AI-2 synthesis [62]. Swarming, a flagella and cell-density dependent phenomenon, is also reduced in *uvrY* delectation mutant and complementation of *uvrY* in the mutant restored swarming in UPEC [31]. This further confirms the integration of the BarA/UvrY/CsrA pathway with AI-2 based quorum sensing signaling and biofilm formation.

This study indicates that the BarA-UvrY-CsrA pathway regulate *E*. *coli* metabolism, nutrient acquisition and cell-cell signaling and forms an underlying basis of bacterium-host signaling recognition and pathogenesis. Because both *luxS* and the BarA/UvrY/CsrA pathway are conserved in several bacterial species, such regulation and integration might be investigated in clinically relevant pathogens. The integration of the BarA/UvrY/CsrA pathway and its homologs with cell-to-cell signaling in diverse pathogenic bacteria may be explored to determine if integration of metabolic pathway with cell-to cell signaling can be viewed as common themes in biological regulation. However, many strains of *E*. *coli* lack a functional *lsr* gene which might lead to accumulation of AI-2 in the extracellular environment and consequently cross species signaling might be more frequent in certain niche for coordinating changes in gene expression [63]. Depending on the polymicrobial environment, the regulatory interactions of the pathway will influence accumulation of AI-2 in the extracellular environment and consequently AI-2 signaling can have varying role in the community structure and dynamics. Targeting two-component regulatory system that integrates with cell-to-cell signaling may influence both intraspecies and interspecies microbial communication in a niche-specific manner. These strategies may be effective for development of novel therapeutics particularly for infections that are difficult to treat due to emergence of antibiotic resistance.

## Supporting Information

S1 Fig AI-2 accumulation in *Escherichia coli* MG1655 (harbouring wild type *luxS* gene), *luxS* deletion mutant and plasmid complemented strains of *luxS* deletion mutant. Note the wild type in this experiment is MG1655, which is not the same as SM1005, a merodiploid strain. Accumulation of AI-2 in the cell-free supernatant was assayed by *Vibrio harveyi* reporter assay as described in the text. Dotted lines indicate growth of corresponding bacterial strains. The graphs represent mean of three experiments. Representative data point used for calculation of significance is shown in asterisk.
(TIF)

S2 Fig Northern analysis of *luxS* message in Δ*barA* or Δ*uvrY* mutant. Relative pixel intensity of the signal to rRNA signal is expressed as numbers at the bottom. Deletion of *barA* or *uvrY* reduced expression of *luxS* whereas complementation of the mutant restored the expression level of *luxS* similar to the wild-type. This experiment was repeated twice.
(TIF)

S3 Fig Effect of *hfq* on *luxS*::*lacZ* reporter activity. Mutation in *hfq* reduced reporter activity at the entry of stationary phase and complementation of Hfq in the mutant restored the expression of *luxS* fusion. This experiment was repeated two times. Representative data point used for calculation of significance is shown in asterisk.
(TIF)

S1 File Data. (XLSX)
