# Supplementary figures and images for: Integration of AI-2 Based Cell-Cell Signaling with Metabolic Cues in Escherichia coli
Source: PLoS One. 2016 Jun 30;11(6):e0157532. doi: 10.1371/journal.pone.0157532 (PMC4928848; doi:10.1371/journal.pone.0157532)

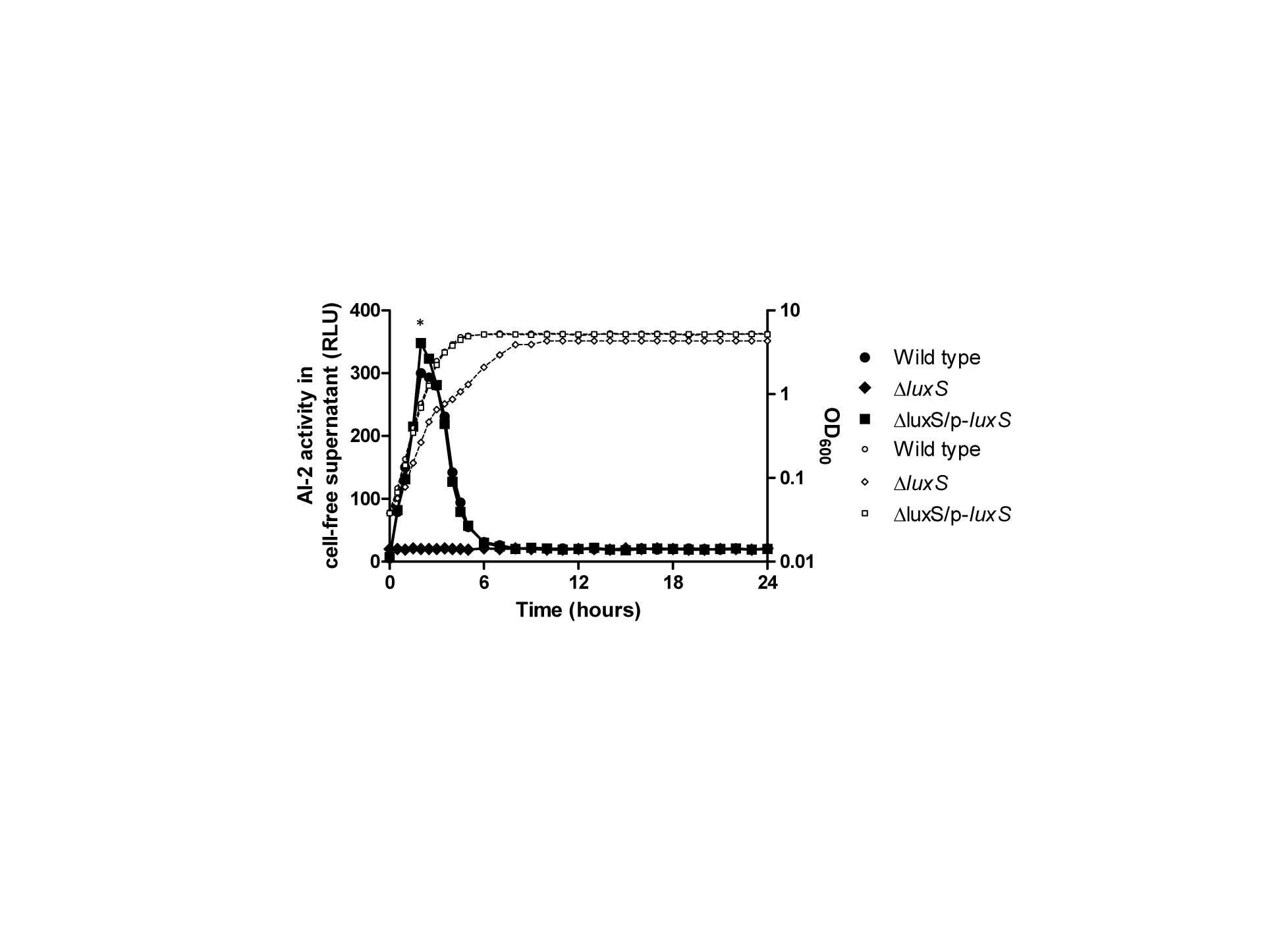

Supplement: S1 Fig — Note the wild type in this experiment is MG1655, which is not the same as SM1005, a merodiploid strain. Accumulation of AI-2 in the cell-free supernatant was assayed by Vibrio harveyi reporter assay as described in the text. Dotted lines indicate growth of corresponding bacterial strains. The graphs represent mean of three experiments. Representative data point used for calculation of significance is shown in asterisk. (TIF) [file pone.0157532.s001.tif]

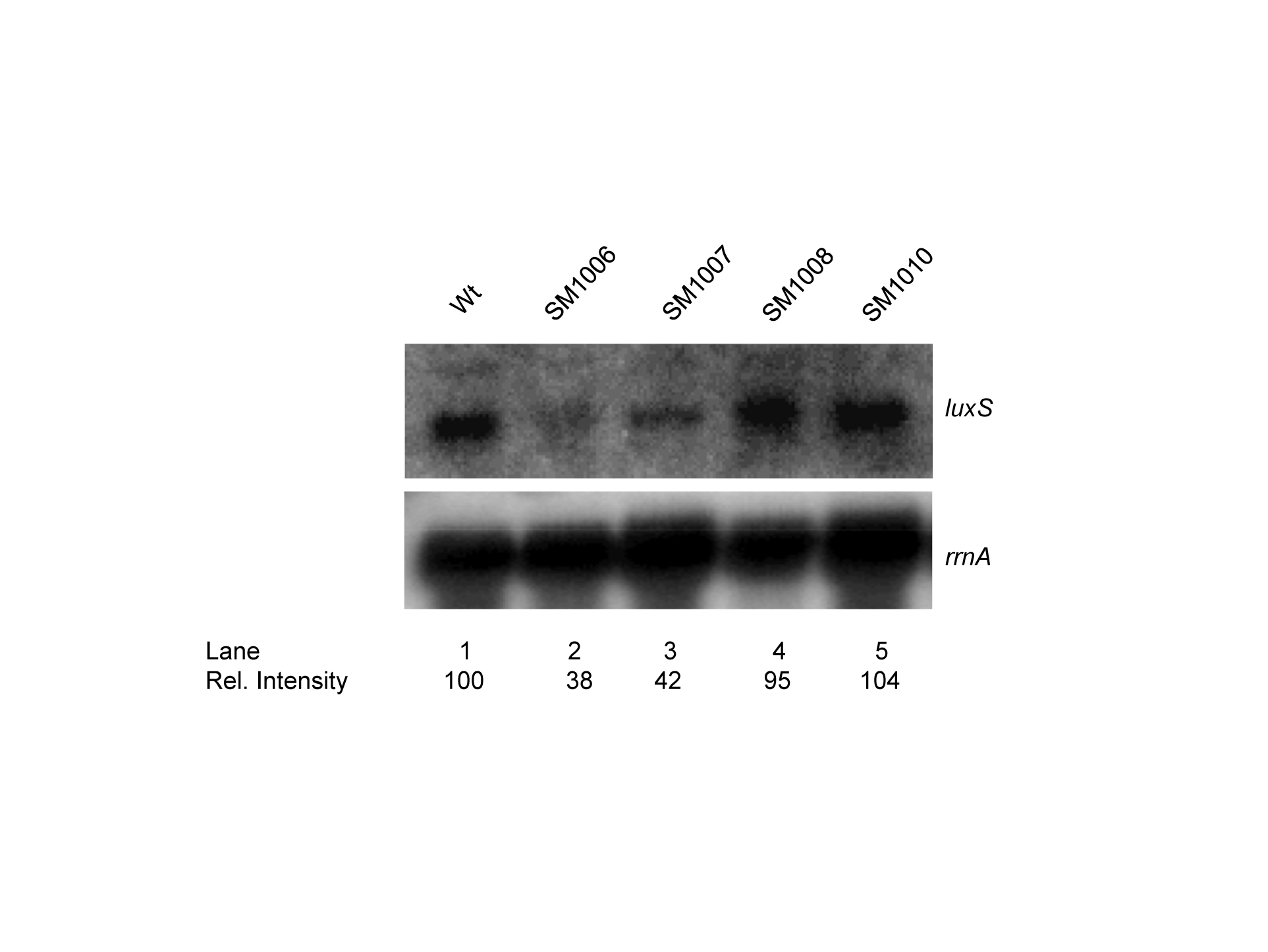

Supplement: S2 Fig — Relative pixel intensity of the signal to rRNA signal is expressed as numbers at the bottom. Deletion of barA or uvrY reduced expression of luxS whereas complementation of the mutant restored the expression level of luxS similar to the wild-type. This experiment was repeated twice. (TIF) [file pone.0157532.s002.tif]

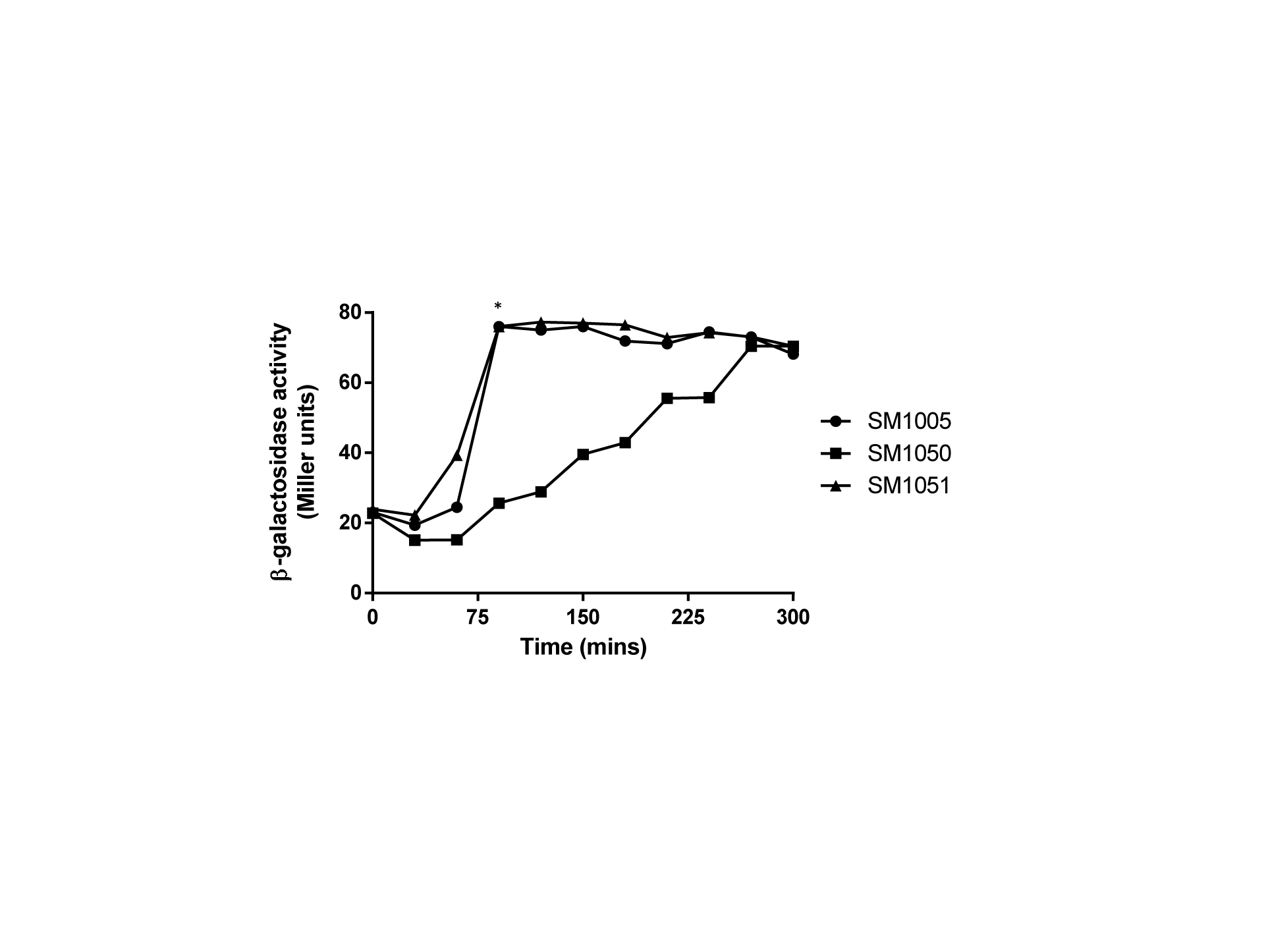

Supplement: S3 Fig — Mutation in hfq reduced reporter activity at the entry of stationary phase and complementation of Hfq in the mutant restored the expression of luxS fusion. This experiment was repeated two times. Representative data point used for calculation of significance is shown in asterisk. (TIF) [file pone.0157532.s003.tif]
